# Small bowel strangulation due to peritoneopericardial diaphragmatic hernia

**DOI:** 10.1186/1749-8090-9-65

**Published:** 2014-04-02

**Authors:** Jang-Hoon Lee, Se-Won Kim

**Affiliations:** 1 Department of Thoracic and Cardiovascular Surgery, College of Medicine, Yeungnam University, Daemyeong 5-dong, Nam-gu, Daegu 705-717, Korea; 2 Department of Surgery, College of Medicine, Yeungnam University, Daegu, Korea

**Keywords:** Diaphragm, Hernia, Surgery, Peritoneopericardial hernia

## Abstract

A 75-year-old Korean man was referred to our hospital with cramping abdominal pain. His chest X-ray showed an abnormal air shadow above the diaphragm, and computed tomography showed an abdominal viscera in the pericardium. We performed surgery and confirmed peritoneopericardial diaphragmatic hernia with small bowel strangulation. Postoperative course was uneventful. Peritoneopericardial diaphragmatic hernia is very rare in humans, so we report the case with a literature review.

## Background

A peritoneopericardial diaphragmatic hernia is one that permits direct communication between the peritoneal and pericardial cavities through a defect in the diaphragm. It is very rare in humans. The cause of peritoneopericardial diaphragmatic hernia varies, as some authors have reported [1-5]. We herein report a case of peritoneopericardial diaphragmatic hernia with small bowel strangulation in a 75-year-old Korean man.

## Case presentation

**Figure 1:**
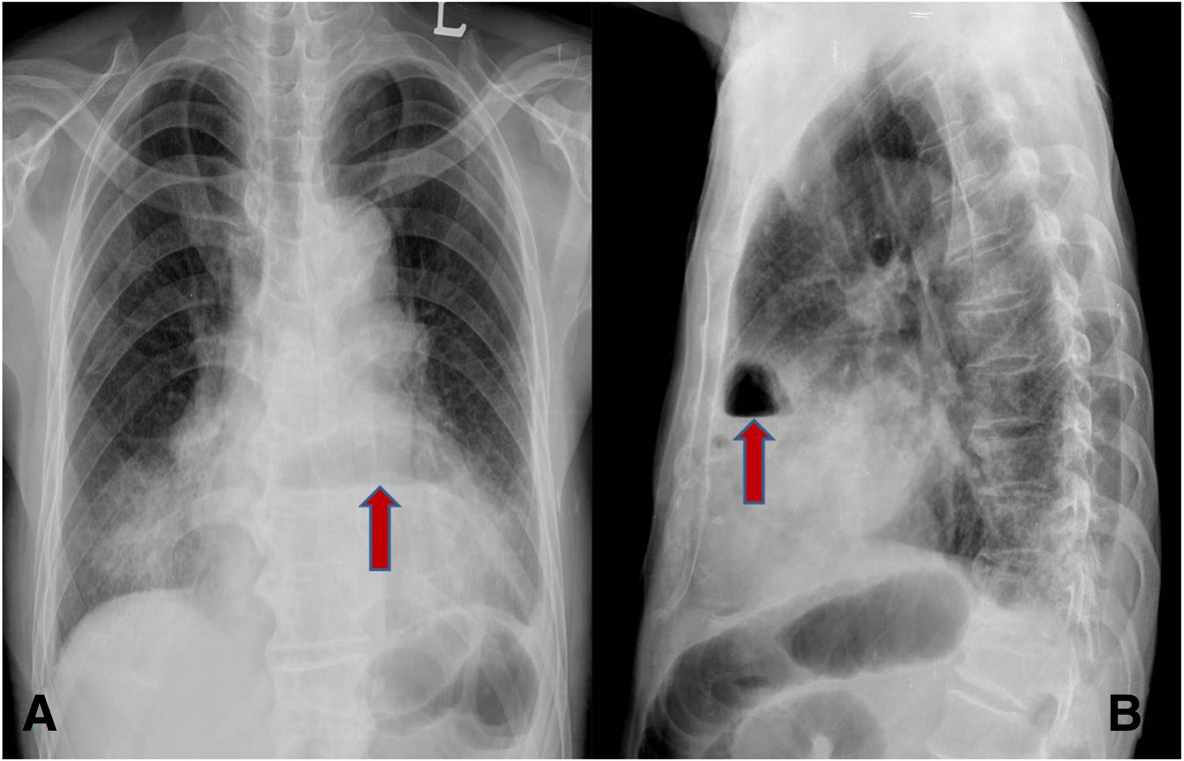
**Preoperative chest radiograph. A**: Chest PA shows an abnormal air shadow above the diaphragm (arrow). **B**: Chest lateral view shows an abnormal air shadow under the sternum.

**Figure 2:**
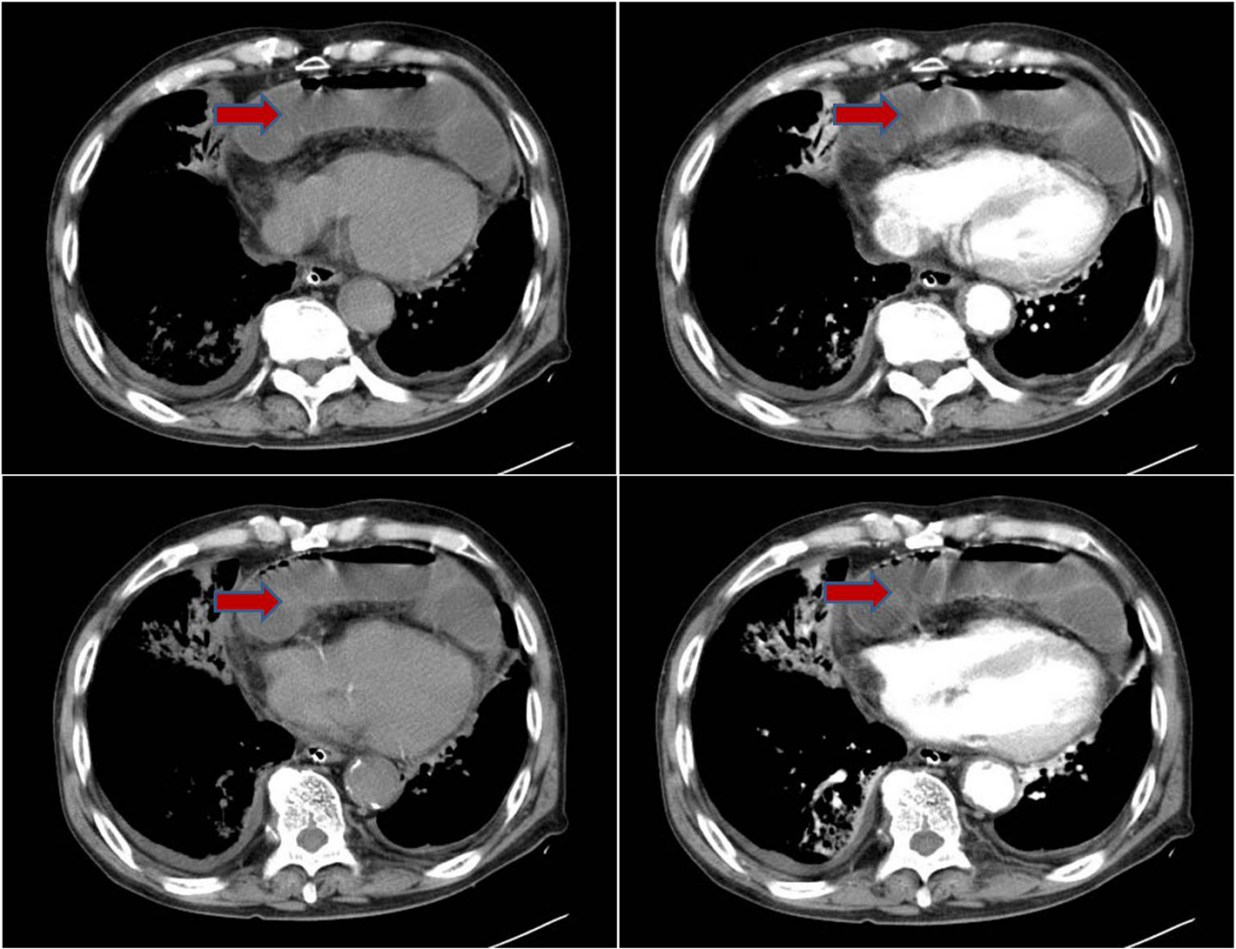
Computed tomography (CT) demonstrates a loop of intestine in the pericardial space (arrow).

**Figure 3:**
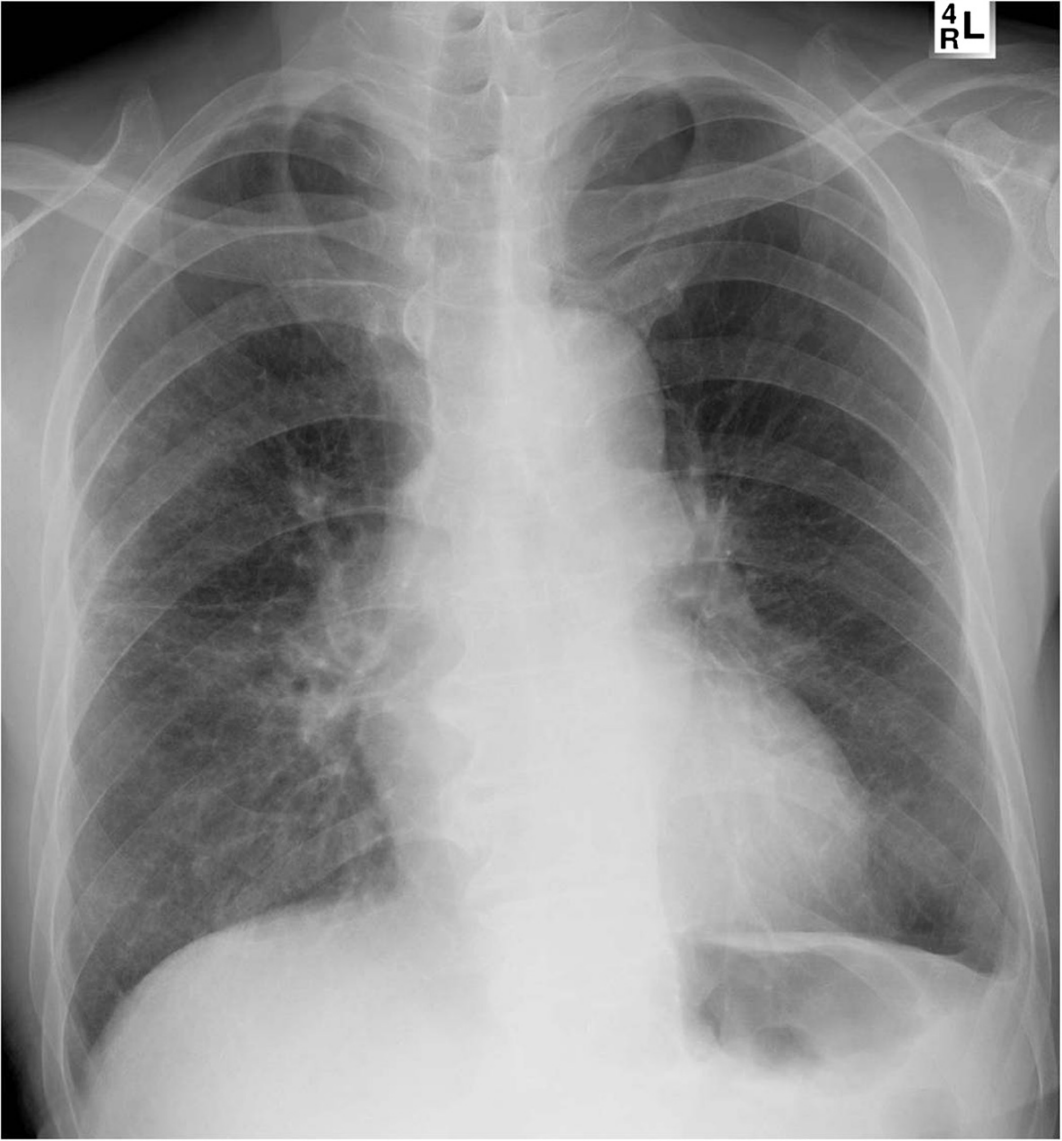
**Postoperative chest x-ray.** Abnormal air shadows observed preoperatively were not seen.

A 75-year-old Korean male patient was referred to our emergency department with cramping abdominal pain for three days. He also had dyspnea and chest discomfort. He had diabetes mellitus and no history of trauma. Upon his arrival at the emergency department, his blood pressure was 150/100 mmHg, heart rate 102 per minute, respiratory rate 22 per minute and body temperature 37.5°C. On physical examination there was direct tenderness on the whole abdomen and we could hear rale in both lower lung fields. Laboratory studies showed leukocytosis and a leukocyte count of 1,500. An x-ray of the chest showed abnormal air shadows above the diaphragm (Figure 1). Computed tomography showed what appeared to be a loop of intestine in the pericardium (Figure 2). We performed an emergency operation with median laparotomy. The small bowel was herniated to the pericardial space through the diaphragmatic defect and the herniated bowel was edematous and strangulated. We pulled the herniated bowel for an easy manual reduction. After the reduction of the herniated bowel, we identified the diaphragmatic defect. The defect was located at the left side of the central tendinous portion and had no communication with the pleural space. The defect was 3 × 3 cm in size with a round shape, and the margin of the defect was smooth and thick. We could see the heart beat through the defect. We resected the strangulated small bowel and performed end-to-end anastomosis. The defect was closed with Gortex patch with interrupted ethibond sutures. On chest x-ray after operation, no bowel gas was seen in the chest (Figure 3). The patient received ventilator care during the immediate postoperative period. On the fourth postoperative day, the patient was transferred to a general ward and the postoperative course was uneventful. The patient was discharged on the ninth postoperative day. We concluded that the patient’s final diagnosis was small bowel strangulation due to peritoneopericardial diaphragmatic hernia.

## Discussion

A peritoneopericardial diaphragmatic hernia is one that permits direct communication between the peritoneal and pericardial cavities through a defect in the diaphragm. It is very rare in humans. Peritoneopericardial hernia may be congenital, arising from the failure of development of the transverse septum [1], or may follow rupture of the diaphragm after trauma [2,3]. A surgical cause has also been reported [4,5]. Since our patient had a defect of the central tendon of the diaphragm with peritoneopericardial communication and no history of trauma, we concluded that the defect was congenital. The signs and symptoms of peritoneopericardial diaphragmatic hernia are dyspnea, cyanosis, palpitation, and chest pain; if viscera become incarcerated, signs and symptoms of intestinal obstruction are added [6]. Cardiac tamponade and pericardial effusions are unusual, but have been reported [7,8]. Our patient had dyspnea, chest discomfort and symptoms of viscera incarceration. When diaphragmatic hernia is suspected, an x-ray of the chest will demonstrate viscera above the diaphragm. The chest x-ray is abnormal in the majority of cases, usually showing air within the pericardium [9]. In 1947, Wilson et al. described the radiographic appearance of the peritoneopericardial diaphragmatic hernia [10]. Our patient had an abnormal chest radiographic appearance that showed abnormal air shadows above the diaphragm and confirmed the computed tomography. The only definitive treatment for peritoneopericardial hernia is surgery. In 1907, Wilson et al. reported the first case successfully corrected with surgery [10]. Some authors suggest that a thoracotomy incision is the only logical surgical approach [6]. But the logical operative approach may vary, depending on the acuity and associated conditions [9]. In the case of acute herniation, in which abdominal viscera injuries present, repair from an abdominal approach is indicated. However, in chronic situations, adhesion to the pericardium and heart are likely to have developed, making exposure through the chest more desirable [9]. Repair procedures for the defect depend on the size of the defect. Primary repair with nonabsorbable suture is satisfactory when the diaphragmatic margins are easily approximated without tension, but a large defect requires patch repair [9]. In our case, the patient had cramping abdominal pain, so we suspected strangulation of the abdominal viscera. We therefore performed median laparotomy. The margin of the defect was thick and not easily approximated, so we performed a patch repair of the defect.

## Conclusions

A case of small bowel strangulation due to peritoneopericardial hernia and successfully corrected surgically is reported. Abnormal air shadow above the diaphragm on chest x-ray led us to suspect a type of diaphragmatic hernia. Computed tomography is one of the best diagnostic methods. The clinician should be aware of peritoneopericardial diaphragmatic hernia, however rare this condition. If peritoneopericardial diaphragmatic hernia is confirmed, surgical repair should be performed.

## Consent

Written informed consent was obtained from the patient for publication of this case report and any accompanying images. A copy of the written consent is available for review by the Editor-in-Chief of this journal.

## Competing interests

The authors declare that they have no competing interests.

## Authors’ contributions

JH and SW wrote the draft of the manuscript and obtained the written consent. JH performed the literature review and participated in the manuscript writing and helped to the final writing of the paper and gave final approval of the manuscript. Both authors have read and approved the final manuscript.
